# Genome-wide analysis of *HSP70* gene superfamily in *Pyropia yezoensis* (Bangiales, Rhodophyta): identification, characterization and expression profiles in response to dehydration stress

**DOI:** 10.1186/s12870-021-03213-0

**Published:** 2021-09-24

**Authors:** Xinzi Yu, Zhaolan Mo, Xianghai Tang, Tian Gao, Yunxiang Mao

**Affiliations:** 1 Key Laboratory of Marine Genetics and Breeding (Ministry of Education), Ocean University of China, Qingdao, 266003 China; 2 College of Marine Life Sciences , Ocean University of China, 5 Yushan Road, Qingdao, 266003 China; 3 Key Laboratory of Utilization and Conservation of Tropical Marine Bioresource (Hainan Tropical Ocean University), Ministry of Education, Sanya, 572022 China; 4 Laboratory for Marine Biology and Biotechnology, Qingdao National Laboratory for Marine Science and Technology, Qingdao, 266237 China

**Keywords:** *Pyropia yezoensis*, *HSP70* gene family, Genome-wide analysis, Dehydration stress, Phylogeny, Gene structure, Expression pattern, Subcellular localization

## Abstract

**Background:**

Heat shock proteins (HSPs) perform a fundamental role in protecting plants against abiotic stresses. Individual family members have been analyzed in previous studies, but there has not yet been a comprehensive analysis of the *HSP70* gene family in *Pyropia yezoensis*.

**Results:**

We investigated 15 putative *HSP70* genes in *Py. yezoensis*. These genes were classified into two sub-families, denoted as DnaK and Hsp110. In each sub-family, there was relative conservation of the gene structure and motif. Synteny-based analysis indicated that seven and three *PyyHSP70* genes were orthologous to *HSP70* genes in *Pyropia haitanensi*s and *Porphyra umbilicalis*, respectively. Most *PyyHSP70s* showed up-regulated expression under different degrees of dehydration stress. *PyyHSP70-1* and *PyyHSP70-3* were expressed in higher degrees compared with other *Pyy*HSP70s in dehydration treatments, and then expression degrees somewhat decreased in rehydration treatment. Subcellular localization showed PyyHSP70-1-GFP and PyyHSP70-3-GFP were in the cytoplasm and nucleus/cytoplasm, respectively. Similar expression patterns of paired orthologs in *Py. yezoensis* and *Py. haitanensis* suggest important roles for *HSP70*s in intertidal environmental adaptation during evolution.

**Conclusions:**

These findings provide insight into the evolution and modification of the *PyyHSP70* gene family and will help to determine the functions of the *HSP70* genes in *Py. yezoensis* growth and development**.**

**Supplementary Information:**

The online version contains supplementary material available at 10.1186/s12870-021-03213-0.

## Background

Heat shock proteins (HSPs) are found in almost all organisms, from bacteria to humans [1]. In plants, members of the family of HSPs act in cell protection through the folding and translocation of nascent proteins and the refolding of denatured proteins under both stress and non-stress conditions [2, 3]. HSPs can be divided into five families based on molecular weight: HSP70, 70-kDa heat shock protein; HSP90 and HSP100 family; HSP60, chaperonin family; and sHSP, small heat shock protein. Of these, HSP70 is widely conserved and has been shown to play roles in development and defense mechanisms under various stresses.

The *HSP70* gene family contains three highly conserved domains: a C-terminal domain about 10 kDa in size that can bind substrate, an intermediate domain 15 kDa in size, and an N-terminal domain (NBD) 44 kDa in size that binds ATP [4]. Plant *HSP70* genes have been localized to four locations: the cell nucleus/cytoplasm, endoplasmic reticulum (ER), plastids, and mitochondria, with different functions in different locations [5, 6]. Deficiency of some cytosolic *HSP70*s led to severe growth retardation, and heat treatment of plants deficient in *HSP70* genes dramatically increases mortality, indicating that cytosolic HSP70s plays an essential role during normal growth and in the heat response by promoting the proper folding of cytosolic proteins [7, 8].

Ectopic expression of a cytosolic *CaHSP70-2* gene resulted in altered expression of stress-related genes and increased thermotolerance in transgenic *Arabidopsis* [9]. Cytosolic HSP70A in *Chlamydomonas* regulates the stability of cytoplasmic microtubules [10, 11]. Transgenic tobacco plants that over-expressed nuclear-localized NtHSP70-1 exhibited decreased fragmentation and degradation of nuclear DNA during heat-/drought-stress [6, 12]. Knockout experiments indicate that the import of stromal HSP70s into the chloroplast stroma is essential for plant development and important for the thermo-tolerance of germinating seeds [13]. Transgenic tobacco plants constitutively expressing elevated levels of BIP (an ER-localized HSP70 homologue) exhibited tolerance to water deficit by preventing endogenous oxidative stress [14]. In rice, the *BIP1*/*OsBIP3* gene, encoding HSP70 in the ER, regulates the stability of XA21 protein to interfere with XA21-mediated immunity [15]. Mitochondrial HSP70 can suppress programmed cell death in rice protoplasts by maintaining mitochondrial membrane potential and inhibiting the amplification of reactive oxygen species (ROS) [16]. However, the biological functions of most HSP70s in nori have not yet been elucidated, partly due to a lack of information about coding genes or other genomic information. *Pyropia yezoensis* (Bangiales, Rhodophyta) is an economically important seaweed that is cultivated in the intertidal zones of China coastlines [17]. The production and quality of the cultivated *Py. yezoensis* thalli are significantly influenced by intertidal environmental stress. Tidal exposure imposes considerable environmental stress on intertidal seaweeds due to altered irradiance levels [18], temperature changes [19], and direct effects from desiccation [20, 21].

In this study, all of the non-redundant members of *HSP70* genes in *Py. yezoensis* were screened from available, high-quality, chromosomal-level genomes. We determined the characteristics of *PyyHSP70* genes based on the physicochemical properties, genomic locations, and conserved motifs, promoters, and analyzed the phylogenetic relationships of these genes. In addition, the expression levels of the *PyyHSP70* genes were analyzed under dehydration and rehydration conditions. Finally, highly expressed PyyHSP70 proteins were localized in *Arabidopsis* protoplasts. Our findings will be useful resources for future studies of the functions of *HSP70* genes in algae, which will help us understand the evolution of *HSP70* genes in different species.

## Results

### Genome-wide identification of *PyyHSP70* genes in* Py. yezoensis*

**Fig.1:**
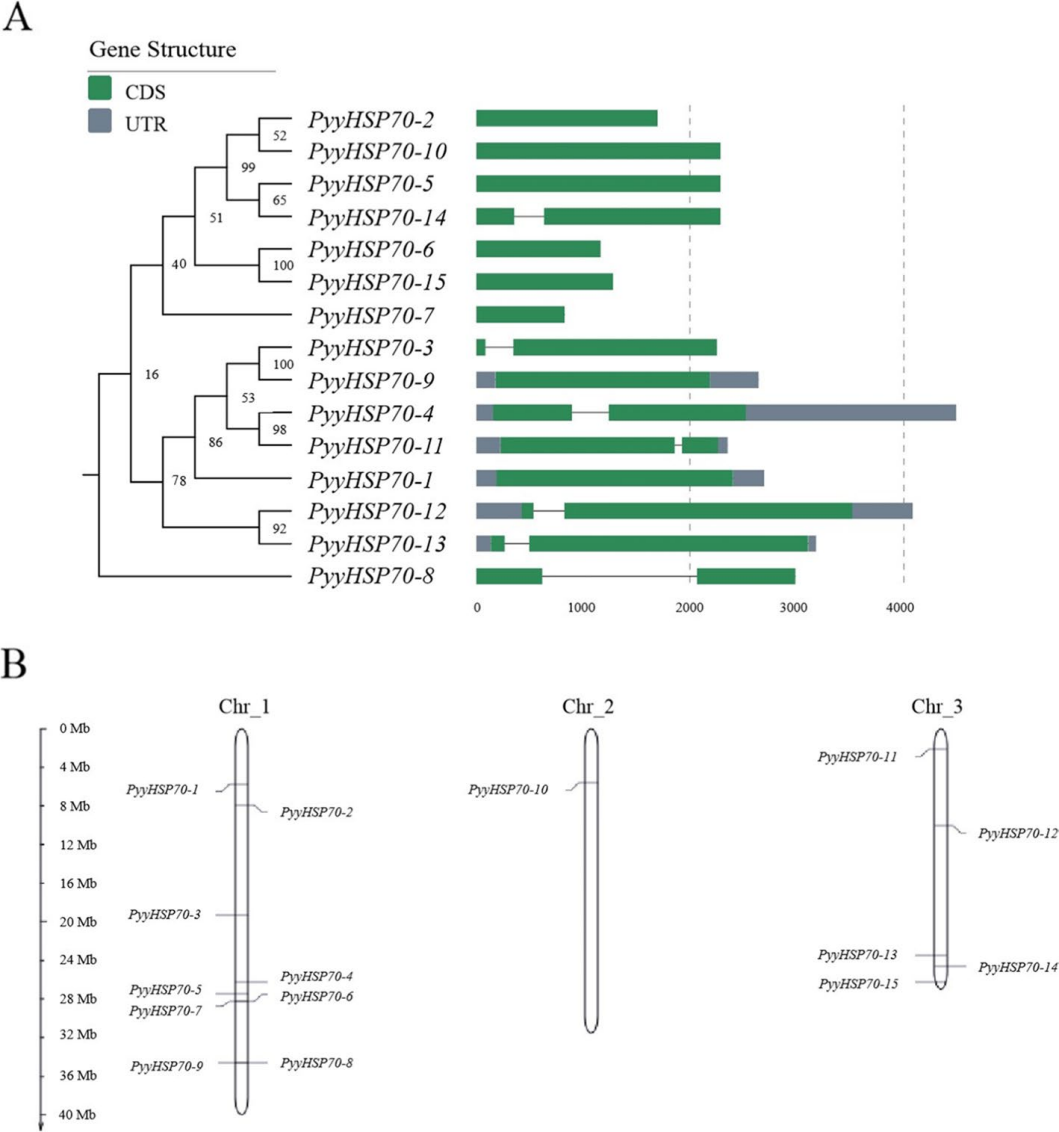
**A** Phylogenetic tree and gene structures of *PyyHSP70* genes. **B** Chromosomal location of *PyyHSP70* genes

After verification, the sequence information was obtained from the *Py. yezoensis* genome for 15 putative *PyyHSP70s.* The basic information of *PyyHSP70* genes (including genomic position, gene length, intron number, amino acid number, isoelectric point (pI), molecular weight, CDS, subcellular localization, and instability index) is listed in Table 1. The predicted PyyHSP70 protein sequences ranged from 276 amino acids to 934 amino acids, and the molecular weights ranged from 29.59 to 96.07 kDa. Analysis with the Expasy online tool revealed instability index values of PyyHSP70s that ranged from 21.76 to 48.25, with a single PyyHSP70 member (PyyHSP70-8) having an instability index greater than 40, indicating an unstable protein. Of the 15 PyyHSP70 proteins, 11 members are predicted to localize to the nucleus/cytoplasm, one to the ER, one to the mitochondria, and two to the chloroplasts. The genes either had no introns or one intron, with eight and seven members respectively (Fig. 1A). The 15 members of *PyyHSP70* were distributed on all three chromosomes, with an uneven distribution in the genome. Chromosome 1 had the highest density of *PyyHSP70* genes, nine members (Fig. 1B).

**Table 1:** List of 15 *HSP70* genes identified in *Py. yezoensis*, including sequence characteristics and subcellular localization

**No**	**Gene ID**	**Chr**	**Location coordinates** **(5’-3’)**	**Gene length (bp)**	**Introns**	**Protein**			**Subcellular location**	**Instability index**
						**Length (aa)**	**pI**	**MW** **(Da)**		
*PyyHSP70-1*	py10939	Chr_1	6,257,822–6,260,514	2693	0	737	5.3	77,016.68	Mitochondria	35.44
*PyyHSP70-2*	py09690	Chr_1	8,661,298–8,659,601	1698	0	567	5.75	60,687.44	Cytoplasm	32.37
*PyyHSP70-3*	py04861	Chr_1	21,055,120–21,057,372	2253	1	664	4.93	71,753.77	Nuc/cytoplasm	32.23
*PyyHSP70-4*	py08976	Chr_1	28,667,875–28,672,367	4493	1	674	5.48	73,556.87	Chloroplast	35.68
*PyyHSP70-5*	py07727	Chr_1	29,911,916–29,909,631	2286	0	762	5.55	81,436.06	Cytoplasm	37.57
*PyyHSP70-6*	py03833	Chr_1	30,853,098–30,851,938	1161	0	387	8.3	42,195.65	Cytoplasm	27.28
*PyyHSP70-7*	py03832	Chr_1	30,853,971–30,853,144	828	0	276	5.45	29,592.44	Nuc/cytoplasm	21.76
*PyyHSP70-8*	py05934	Chr_1	37,681,333–37,684,320	2988	1	514	6.54	49,216.21	Nuc/cytoplasm	48.25
*PyyHSP70-9*	py05935	Chr_1	37,688,796–37,686,154	2643	0	669	5.17	72,123.3	Cytoplasm	36.36
*PyyHSP70-10*	py01115	Chr_2	6,095,554–6,097,839	2286	0	762	5.58	81,548.18	Cytoplasm	36.61
*PyyHSP70-11*	py07959	Chr_3	2,333,040–2,330,689	2352	1	658	5.16	70,389.67	Chloroplast	38.56
*PyyHSP70-12*	py03715	Chr_3	10,900,154–10,896,070	4085	1	934	4.92	96,065.46	Cytoplasm	34.16
*PyyHSP70-13*	py11056	Chr_3	25,538,961–25,535,784	3178	1	914	5.62	92,825.4	ER	39.52
*PyyHSP70-14*	py08919	Chr_3	26,793,406–26,795,692	2287	1	670	5.44	71,561.76	Cytoplasm	34.2
*PyyHSP70-15*	py09058	Chr_3	28,567,799–28,569,082	1284	0	428	8.71	47,261.88	Cytoplasm	32.99

### Conserved motifs and phylogenetic analysis of PyyHSP70s

To better understand the structural characteristics of PyyHSP70 proteins, a multiple sequence alignment was performed of the HSP70 domains of all 15 PyyHSP70 proteins and the EcDNAK protein, as shown in Figure S1. The two functional domains (ATPase domain and peptide-binding domain) were present in all PyyHSP70s. The ATPase domains of PyyHSP70-6, PyyHSP70-7*,* and PyyHSP70-15 were shorter, and lacked three signature motifs that are characteristic of the ATPase domain of HSP70 family members (Table S1). Additionally, the peptide-binding domain of PyyHSP70-2 was shorter, and much shorter C-terminal sub-domains were present in PyyHSP70-2 and PyyHSP70-7 (Table S1).

**Fig. 2:**
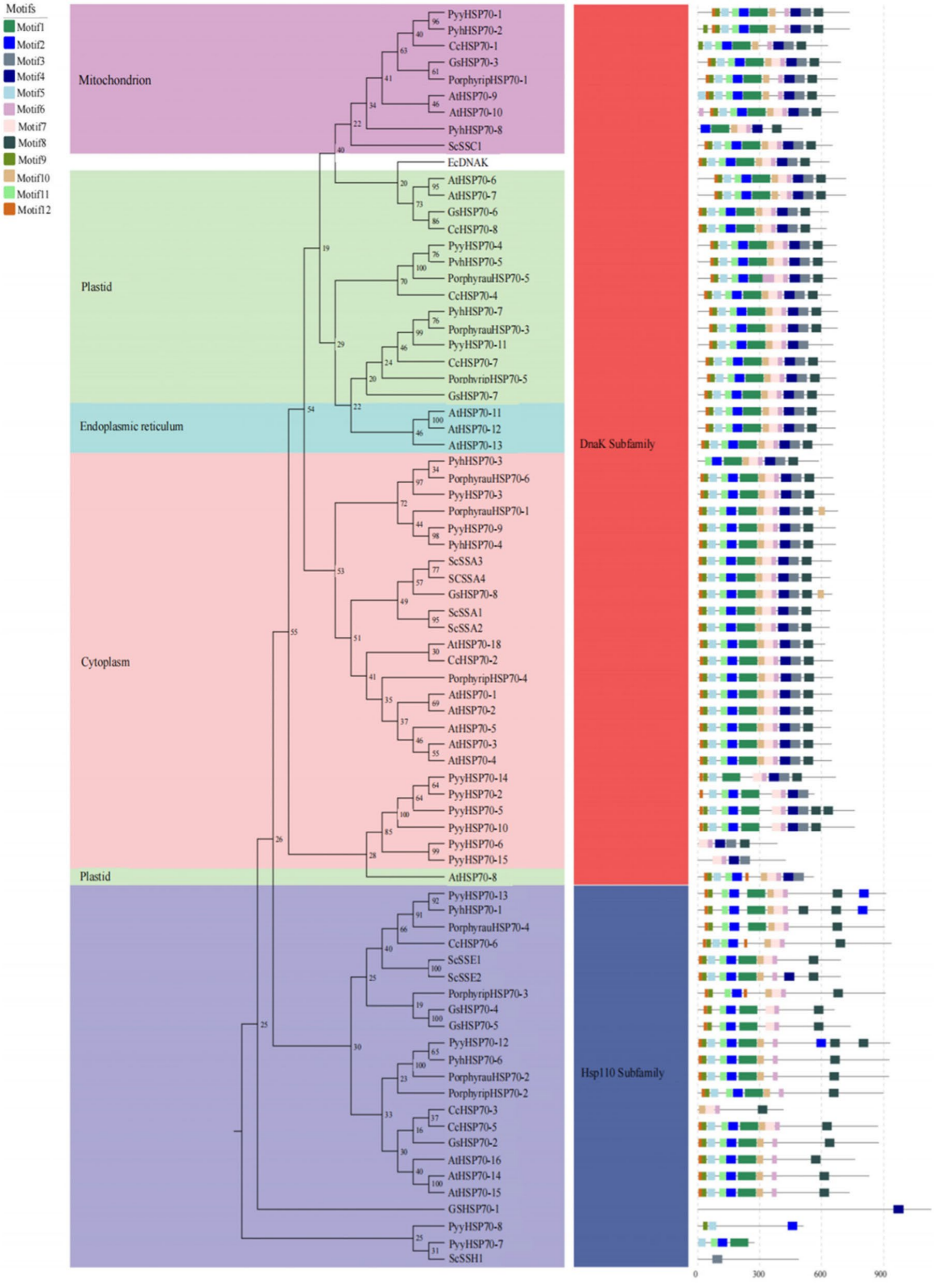
Phylogenetic (left) and conserved motif (right) analysis of HSP70 proteins. Motif analysis was performed using the online MEME program. Different colors of boxes represent different motifs in the corresponding positions of each protein

Twelve consensus motifs were found in PyyHSP70 proteins using the MEME motif search tool (Fig. 2, Table S2). Motifs 1, 2, 5, 6, 7, 9, 10, 11, and 12 were identified in the ATPase domain, and motifs 3, 4, and 8 were identified in the peptide-binding domain. Only motifs 3, 4, 6, and 7 were detected in all PyyHSP70 members of the DnaK subfamily, and only motif 2 was detected in all PyyHSP70 members of the Hsp110 subfamily.

An unrooted phylogenetic tree was constructed to visualize the evolutionary relationships between HSP70 members, using 76 HSP70 protein sequences from nine species (Table S3). As shown in Fig. 2, these HSP70s were classified into two subfamilies (the DnaK subfamily and the Hsp110 subfamily). The DnaK subfamily was further divided into four groups based on localization (cytoplasm, ER, mitochondria, and plastid). The HSP70 proteins from different species were more closely related to those in the same subfamily than to others in the same species. For example, cytosolic PyyHSP70-4 was more closely to PyhHSP70-5 than PyyHSP70-11.

**Fig. 3:**
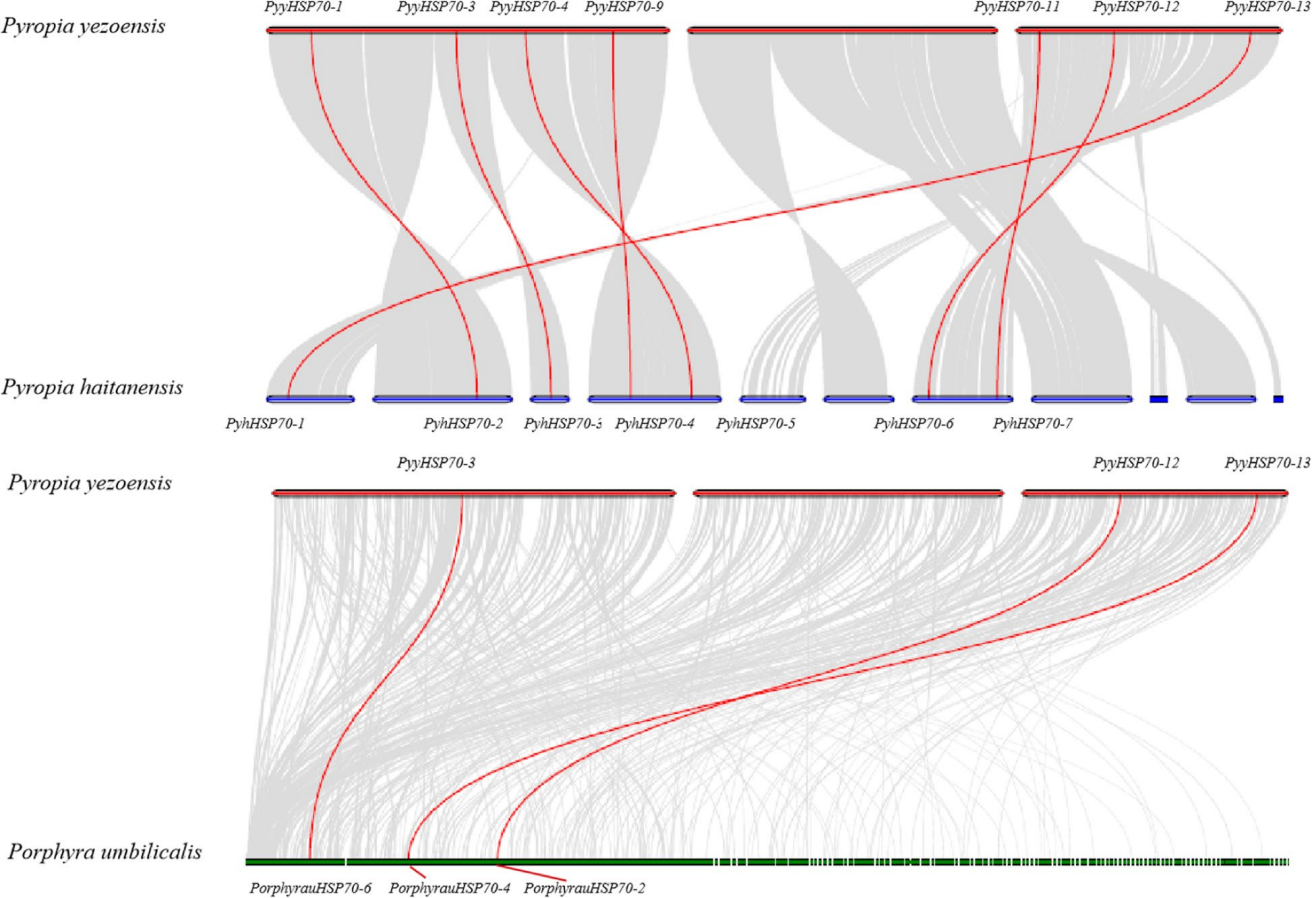
Synteny-based analysis of *HSP70* genes of *Py. yezoensis* and two representative red algae species. Gray background lines indicate collinear blocks within *Py. yezoensis* and other red algae genomes, and the red lines indicate syntenic *HSP70* gene pairs

For the *PyyHSP70* family members, orthologs from *Py. yezoensis* and *Py. haitanensis* (seven pairs) or *Porphyra umbilicalis* (three pairs) were identified, indicating there may have been common ancestral genes of the *HSP70* family before differentiation of the three species (Fig. 3). In addition, a subclade of six genes (*PyyHSP70-2*, *PyyHSP70-5*, *PyyHSP70-6*, *PyyHSP70-10*, *PyyHSP70-14* and *PyyHSP70-15*) in the cytoplasm group implied the proximity of these sequences and potential paralogous relationships by duplication events after the divergence of the two *Pyropia* species. The Ka/Ks ratios of the parolog pairs in the subclade were calculated and the results ranged from 0.6327–1.3487 (Table 2). The Ka/Ks ratios for five pairs were less than but close to one, indicating slightly negative selection; the other two pairs’ Ka/Ks ratios were greater than one, suggesting positive selection. All seven orthologous events of *Pyropia* exhibited Ka/Ks ratios far less than 1 (Table S4).

**Table 2:** Ka, Ks, and Ka/Ks values for duplicated paralog pairs in *Py. yezoensis*

Seq 1	Seq 2	Ka	Ks	Ka/Ks
*PyyHSP7-2*	*PyyHSP7-10*	1.0282	0.9313	1.1040
*PyyHSP7-5*	*PyyHSP7-10*	0.0284	0.0448	0.6327
*PyyHSP7-5*	*PyyHSP7-2*	0.9768	1.0797	0.9048
*PyyHSP7-5*	*PyyHSP7-14*	0.9766	1.0596	0.9217
*PyyHSP7-6*	*PyyHSP7-15*	0.9664	1.1183	0.8642
*PyyHSP7-14*	*PyyHSP7-2*	0.9569	1.1361	0.8423
*PyyHSP7-14*	*PyyHSP7-10*	1.0968	0.8133	1.3487

### Cis-regulatory element analysis of the *PyyHSP70* gene family

The regulatory roles of the identified *PyyHSP70* genes were further studied by analysis of the 2000 bp region upstream of these genes. We searched the promoter sequences using the PlantCARE tool for seven regulatory elements previously found to be involved in various stresses: ABRE, CGTCA-motif, TGACG-motif, TCA-element, MYB-binding sites (MBS), LTR, and DRE (Table 3). Fourteen of these genes possessed several ABRE elements (all but *PyyHSP70-4*); all *PyyHSP70* genes contained the CGTCA-motif and MYB-binding sites (MBS); 13 of 15 *PyyHSP70* genes contain TGACG-motifs (all but *PyyHSP70-13* and *PyyHSP70-15*); 12 of 15 *PyyHSP70* genes contain CRE/DRE elements (all except *PyyHSP70-2, PyyHSP70-4,* and *PyyHSP70-13*). Besides, we also searched three types of heat shock elements (HSEs), perfect type (nTTCnnGAAnnTTCn), gap type (nTTCnnGAAnnnnnnnTTCn) and step (S) type (nTTCnnnnnnnTTCnnnnnnnTTCn) [22], in these promoter sequences. Only one S-type HSE was detected in *PyyHSP70-3.* The detection of these abiotic response elements suggests that the *PyyHSP70* genes may be extensively involved in stress responses, thereby increasing the range of mechanisms that organisms could emply to escape or better cope with adverse environmental effects.

**Table 3:** Summary of stress-inducible cis-elements in the promoter regions of *PyyHSP70* genes

No	ABRE^a^	CGTCA-motif^b^	TGACG-motif^c^	TCA-element^d^	MBS^e^	LTR^f^	DRE^g^
*PyyHSP70-1*	4	6	5	0	1	0	2
*PyyHSP70-2*	9	2	5	0	6	0	0
*PyyHSP70-3*	3	3	2	0	1	1	1
*PyyHSP70-4*	0	3	2	0	9	0	0
*PyyHSP70-5*	11	3	6	0	6	0	1
*PyyHSP70-6*	4	2	7	0	3	0	3
*PyyHSP70-7*	1	1	6	1	3	0	2
*PyyHSP70-8*	6	4	3	0	5	0	4
*PyyHSP70-9*	10	3	2	0	4	1	5
*PyyHSP70-10*	9	4	6	0	4	0	1
*PyyHSP70-11*	2	3	4	0	5	0	2
*PyyHSP70-12*	8	1	5	0	3	0	4
*PyyHSP70-13*	5	2	0	0	2	0	0
*PyyHSP70-14*	9	7	5	0	9	1	1
*PyyHSP70-15*	5	2	0	0	3	0	1

^a^ cis-acting element involved in abscisic acid response

^b^ cis-acting regulatory element involved in MeJA-response

^c^ cis-acting regulatory element involved in the MeJA-response

^d^ cis-acting element involved in salicylic acid response

^e^ MYB binding site involved in drought-inducibility

^f^ cis-acting element involved in low-temperature response

^g^ cis-acting element involved in dehydration, low-temperature, and salt stresses

### Expression patterns of *PyyHSP70* genes under dehydration treatments

**Fig. 4:**
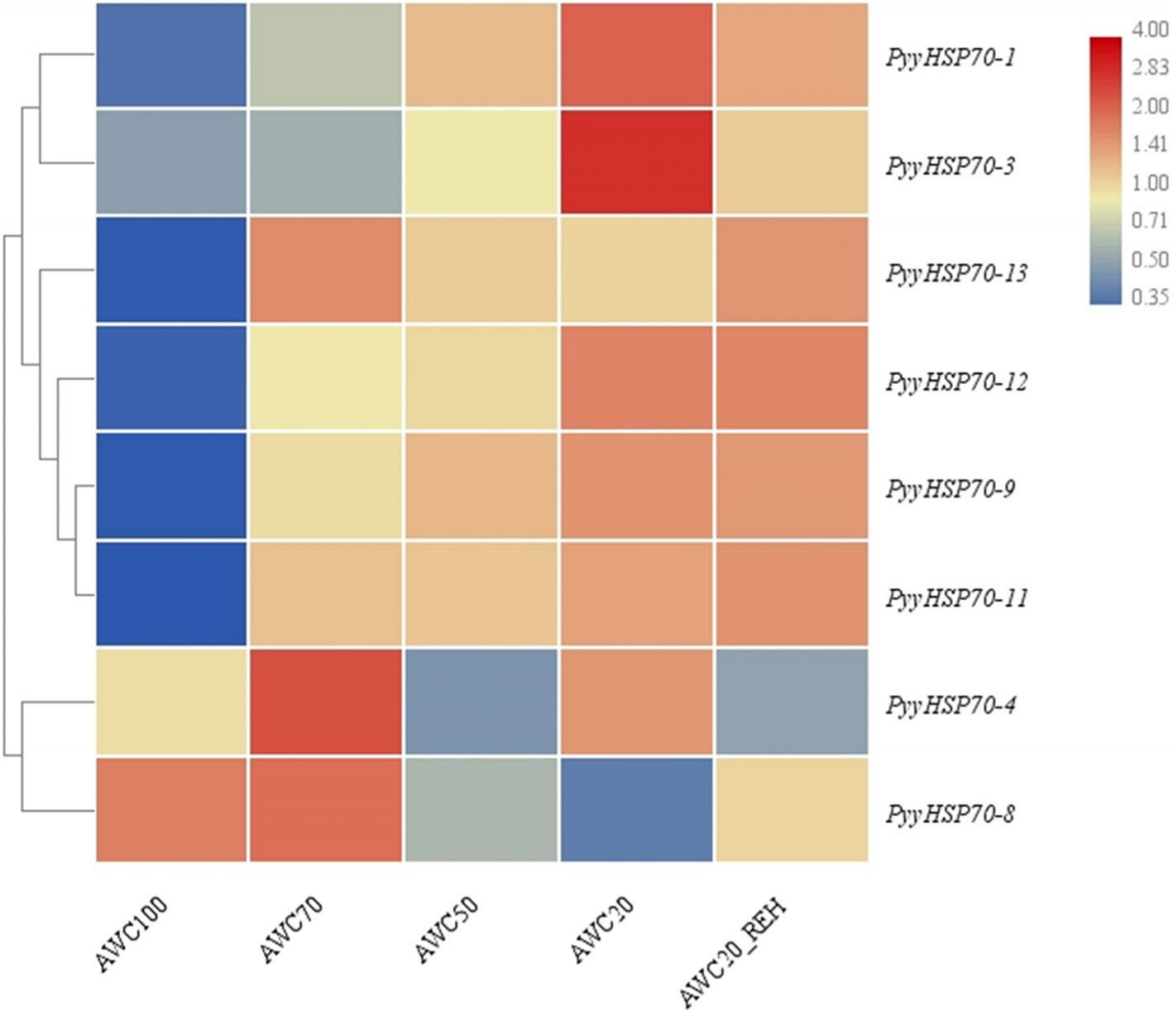
Heatmap of the expression patterns of *PyyHSP70* genes under dehydration and rehydration treatments: absolute water content 100% (AWC100, control), absolute water content 70% (AWC70), absolute water content 50% (AWC50), absolute water content 20% (AWC20), rehydrated 30 min after 20% of water loss (AWC20_30min). The color bar (right) represents log_2_ expression levels (FPKM). The tree (left) represents clustering result of *PyyHSP70*s’ expression patterns

**Fig. 5:**
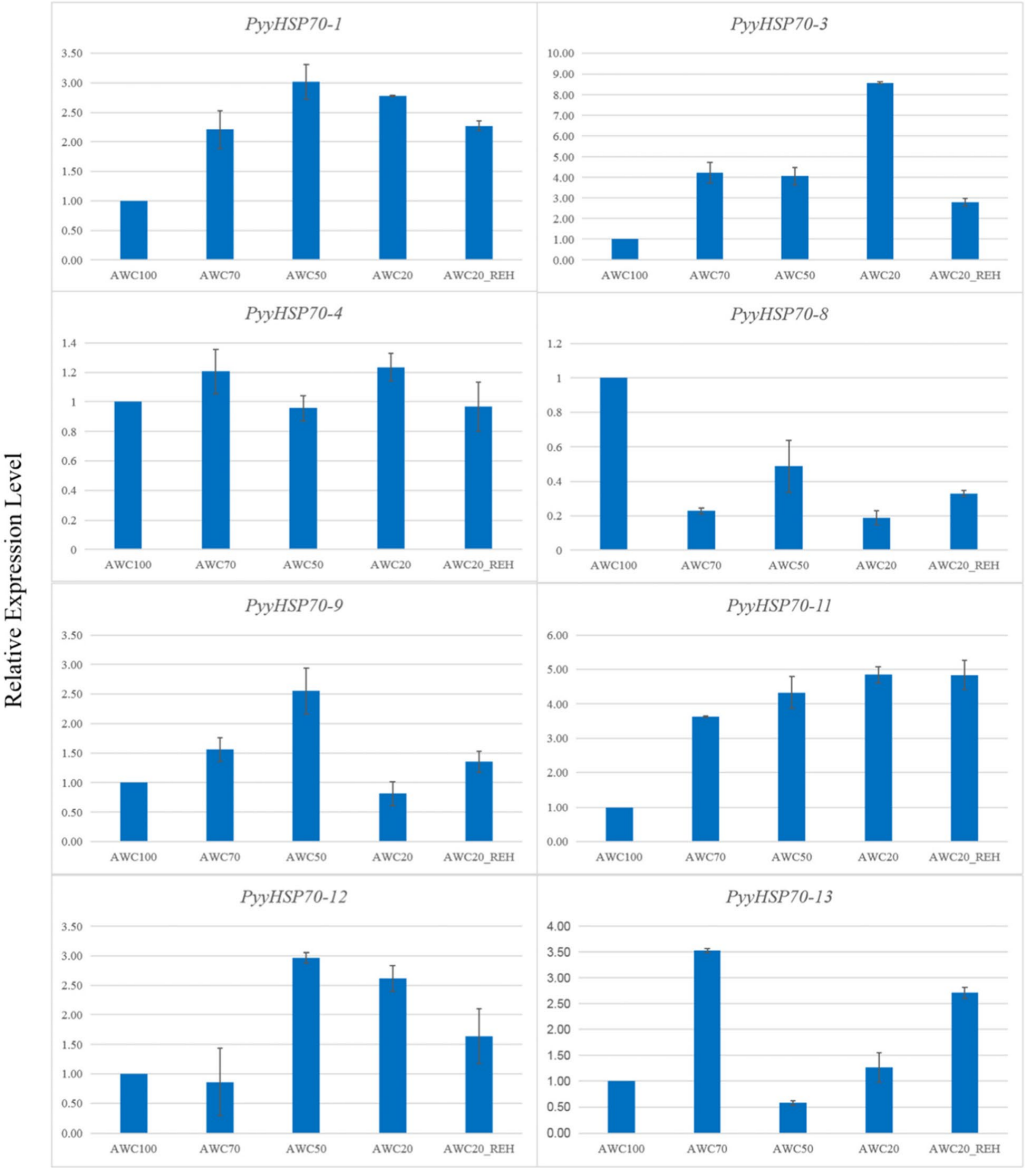
Expression analysis of *PyyHSP70*s under different dehydration stress conditions determined by qRT-PCR. The Y-axis indicates the relative expression level and the X-axis represents different absolute water contents (AWC) of dehydration stress treatments sampled for expression analysis. Each data point represents mean value ± standard deviation (SD) (*n* = 3)

To further clarify the potential ability of the *PyyHSP70* genes to respond to dehydration stress, RNA-Seq data were analyzed. Expression analysis of *PyyHSP70* under dehydration stress revealed low (< 0.3) or no expression from seven genes in all treatments, but the other eight *PyyHSP70* genes exhibited higher expression (Fig. 4). The expression of *PyyHSP70-1* and *PyyHSP70-3* gradually increased with increased dehydration stress, and the expression level slightly decreased with subsequent rehydration treatment. The expression levels of *PyyHSP70-11* increased with increased water loss and continued to increase during rehydration. The expression levels of *PyyHSP70-8*, *PyyHSP70-4*, and *PyyHSP70-13* first increased and then decreased as the degree of dehydration deepened. The expression levels of *PyyHSP70-8* and *PyyHSP70-13* increased during rehydration, and *PyyHSP70-4* experienced an increase of expression for one dehydration condition (AWC20) and then decreased during rehydration. These RNA-seq expression patterns were verified by detecting the expression patterns of the *PyyHSP70* genes by qRT-PCR (Fig. 5). The measured expression levels of most genes were highly consistent with the levels determined by RNA-seq, except for *PyyHSP70-13*.

### The subcellular localization of PyyHSP70-1 and PyyHSP70-3 proteins

**Fig. 6:**
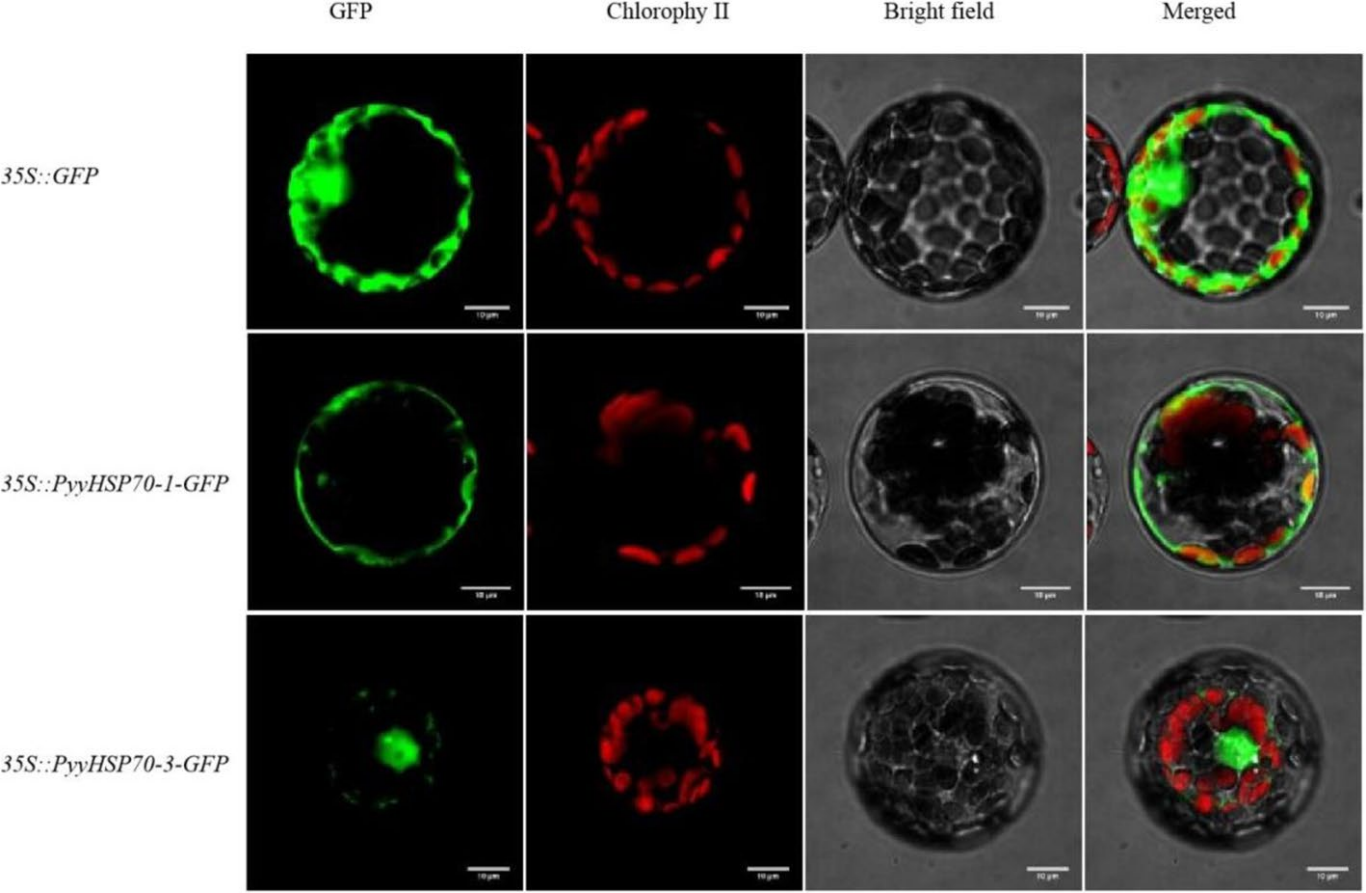
Subcellular localization of PyyHSP70-1 and PyyHSP70-3 in *Arabidopsis* protoplasts. Scale bars = 10 μm. 35S::GFP was used as a negative control

*PyyHSP70-1* and *PyyHSP70-3* showed the biggest expression changes in response to dehydration stress, so we next determined the subcellular localization of these two proteins. PyyHSP70-1 was localized to the cytoplasm, and PyyHSP70-3 localized to the nucleus/cytoplasm (Fig. 6), basically consistent with the predicted results (Table 1, Fig. 6).

## Disscussion

Daily changes in tide height cause air exposure to seaweed, triggering rapidly-changing physical stresses such as dehydration, high temperature, and different irradiance levels [23]. Because they live in the challenging habitat of the intertidal zone, intertidal macroalgae have adapted a set of protective mechanisms to survive [24]. Some intertidal seaweeds are highly tolerant to desiccation. Species of the genera *Pyropia* and *Porphyra* (Bangiales, Rhodophyta) inhabit the upper intertidal zone and can lose up to 95% of cellular water content during maximum low tide [18]. *HSP70* is a superfamily of molecular chaperones widely distributed in eukaryotic cells. These proteins play important roles under abiotic stress by participating in many protein folding processes. However, the *HSP70* superfamily of *Py. yezoensis* was not previously characterized. In this work, we comprehensively analyzed the characteristics, expression patterns under dehydration stress, and the subcellular localization of PyyHSP70s.

### Evolution analysis of *HSP70* genes

In this study, we identified 15 *HSP70* domain-containing genes in the *P. yezoensis* genome that constitute the *HSP70* superfamily, including 11 DnaK subfamily genes and 4 Hsp110 subfamily genes. We also analyzed the genomes of five other red algae and identified 36 *HSP70* genes. We found no direct relationship between genome size and the number of *HSP70* genes in red algae. For example, we identified eight *HSP70* genes in *Py. haitanensis* (genome size: 53 Mb), eight genes in *Galdieria sulphuraria* (genome size: 14 Mb), and eight genes in *Chondrus crispus* (genome size: 105 Mb). This diversity in the number of red algae *HSP70* genes indicated that the *HSP70* gene family has utilized different evolutionary strategies in different species.

*PyyHSP70*s proteins were divided into two sub-families, similar to those reported by previous analysis of *HSP70*s in *A. thaliana* and yeast [5, 25]. The DnaK subfamily was further divided into four groups based on localization. The number of *HSP70* genes from the six red algae was basically same in each group of the DnaK subfamily, except the Cytoplasm group which contained more members of the *PyyHSP70* gene family due to paralogous duplication events. Paralogous duplication events were not evident in the other five red algae, further implying that *PyyHSP70*s expanded according to species-specific approaches during evolution. We found no expression for these paralogous *PyyHSP70* genes with intact gene structures in dehydration treatments, and also no expression of these genes was detected in response to other abiotic/biotic stresses of *P.yezoensis* [20, 21, 26]. *HSP* genes in other species were previously identified that also did not appear to be expressed under tested conditions, but the reason has not yet been determined [27, 28]. Interesting, two-pairs of *PyyHSP70* paralogs showed positive selection, suggesting new functions that should be verified by further experiments.

### *HSP70* genes play essential roles in response to dehydration stress

Previous studies have found abundant HSEs in the promoter regions of *HSP70* genes that become active in response to heat shock and other temperature treatments in higher plants [2, 29]. However, we found few HSE and LTR in the promoter regions of *PyyHsp70* genes. *Py. yezoensis* live on intertidal rocks, where they experience repeated cycles of dehydration and rehydration. *Cis*-regulatory element analysis showed that most *PyyHSP70* gene promoter sequences contained *cis*-elements associated with dehydration stress. For example, the ABRE motif conserved in drought response genes [30, 31], MYB binding sites (MBS) involved in drought-inducibility, and CRT/DRE elements associated with dehydration and salt stresses [32, 33]. The results suggest that *PyyHSP70*s might be significantly related to dehydration response.

Intermittent desiccation stress caused by tidal changes is a significant abiotic factor affecting intertidal seaweed species. This stress can affect the physiology of organisms, mainly through oxidative stress causing destabilization of proteins, leading to loss of membrane integrity [34–36]. Desiccation results in increased expression of tolerance genes, such as genes encoding HSPs and related transcriptional factors [37, 38]. These mechanisms may also function in intertidal seaweed to tolerate desiccation. Several HSP70s have also been found to help protect against desiccation damage by assisting protein-folding processes involved in stress and affecting the proteolytic degradation of unstable proteins [39]. HSP70s have also received attention in marine organisms as a kind of biomarker of stress, because their expression is highly variable in the presence or absence of stimuli [40–42]. Zhou et al. (2011) suggested that analysis of *HSP70* genes could be utilized to evaluate algae tolerance to stresses and monitor coastal environmental changes [43]. Tang et al. (2016) found that moss plants overexpressing PpcpHSP70-2 highly induced by dehydration treatment showed dehydration tolerance [44]. We found that more than half of *PyyHSP70* genes exhibited increased transcription levels with increasing degree of dehydration. We also found significantly increased expression of some *PyyHSP70* genes, especially *PyyHSP70-1* and *PyyHSP70-3,* upon reaching a water content of 20%, with down-regulated expression after rehydration. This finding was consistent with that of a previous study that showed that *HSP70*s played important roles only in the response to extreme desiccation stress [41].

Like *Py. yezoensis*, *Py.haitanensis* also lives in the intertidal zone and experiences repeated dehydration and rehydration (though at a different temperature). These two species are evolutionarily very close, both belonging to *Pyropia*. Paired orthologs between *Py.yezoensis* and *Py.haitanensis* showed strong purifying selection and similar trends in expression (Table S4, Figure S2), suggesting that these *HSP70* orthologs play important roles in dehydration treatments of laver. Therefore, it is important to study the *HSP70* genes involved in the dehydration-induced response of *Py. yezoensis* to further explain the stress resistance and environmental adaptation of intertidal algae.

## Conclusions

The *Py. yezoensis* genome contains 15 members of the *HSP70* gene family, and these genes are unevenly distributed on three chromosomes. The gene structures and phylogenetic analysis suggest a complex evolution history of this gene family in *Py. yezoensis*. The analysis reveals that the *PyyHSP70* family has experienced gene duplication events after species divergence relative to other red algae. Most *HSP70*s showed up-regulated expression under different degrees of dehydration stress, especially *PyyHSP70-1* and *PyyHSP70-3* which showed much higher expression levels in dehydration treatments and slightly decreased expression after rehydration treatment. Similar expression trends of orthologs of *Py.yezoensis* and *Py.haitanensis* in dehydration treatments demonstrate the important roles of these proteins in intertidal environmental adaptation during evolution. PyyHSP70-1-GFP and PyyHSP70-3-GFP were localized in the cytoplasm and the nucleus/cytoplasm, respectively. This overview of this gene family should facilitate further studies of the *HSP70* gene family, particularly in regards to their evolutionary history and biological functions.

## Methods

### Genome-wide identification of *HSP70* proteins in *Py. yezoensis*

The *Py. yezoensis* genome and protein sequences were deposited to DDBJ/ENA/GenBank as accession WMLA00000000 [17]. To identify candidate *Py. yezoensis* HSP70 protein sequences, the Hidden Markov model (HMM) profile of the HSP70 domain was downloaded from the Pfam (http://www.sanger.ac.uk/Software/Pfam/) database (Pfam:PF00012) and then submitted as a query in a HMMER (e-value < 1e^−5^) search (https://www.ebi.ac.uk/Tools/hmmer/) of the *Py. yezoensis* protein database. The obtained protein sequences were screened and verified for the presence of the HSP70 domain using SMART (http://smart.embl-heidelberg.de/) tools [45], CDD (http://www.ncbi.nlm.nih.gov/Structure/cdd/wrpsb.cgi) and InterProScan (http://www.ebi.ac.uk/interpro/result/InterProScan/). The same process was used to obtain the other four red algae *HSP70* family genes from their genome databases [46–49]. For the *PyyHSP70* genes, we determined the chromosomal locations, genomic sequences, full coding sequences, protein sequences, and the sequence of the 2000 nucleotides upstream of the translation initiation codon. The molecular weight (Da) and isoelectric point (pI) was calculated for each gene using the Compute pI/Mw tool from ExPASy (http://www.expasy.org/tools/) [50]. The subcellular localization of proteins was determined by analysis of the WoLF PSORT, Predotar, PSORT, SherLoc2, CELLO, and Softberry databases, and decided based on consensus localization for two or more algorithms. Schematic images of the chromosomal locations of the *PyyHSP70* genes were generated using MapGene2Chrom software (http://mg2c.iask.in/mg2c_v2.1/), according to the chromosomal position information in the NCBI database.

### Gene structure analysis and identification of conserved motifs

To investigate the diversity and structure of members of the *PyyHSP70* gene family, we compared the exon/intron organization of the cDNA sequences and the corresponding genomic DNA sequences of *HSP70* using EVOLVIEW (https://evolgenius.info//evolview-v2/). In addition, the amino acid sequences were subjected to “predict the domain and motif analyses” online with MEME (http://meme-suite.org) [51]. The parameters were as follows: number of repetitions, any; maximum number of motifs, 12; and optimum motif widths, 2 to 300 amino acid residues.

### Multiple alignment and phylogenetic analysis

We constructed two phylogenetic trees, one with only PyyHSP70 protein sequences and the other including 76 HSP70 protein sequences from different species. The gene and protein sequences of *Arabidopsis thaliana*, *Escherichia coli* and yeast were acquired from previous studies [2, 25, 52, 53] and accession GCA_008690995.1 (NCBI). Multiple sequence alignment to full predicted HSP70 protein sequences was performed with Muscle in Molecular Evolutionary Genetics Analysis (MEGA) 7.0 software using default parameters [54]. Sequence alignments were performed with ClustalX software [55]. Phylogenetic trees were constructed using MEGA7.0 with the Neighbor-Joining (NJ) method, and a bootstrap analysis was conducted using 1000 replicates with pairwise gap deletion mode.

### Gene duplication and Ka/Ks analysis

The microsynteny between *Py. yezoensis*, *Py. haitanensis*, and *Po. umbilicalis* was analyzed by MCScanX with the default parameters [56]. The criteria used to analyze potential gene duplications included: (1) the length of the sequence alignment covered ≥ 70% of the longer gene; and (2) the similarity of the aligned gene regions ≥ 70% [57]. Non-synonymous (Ka) substitution and synonymous (Ks) substitution were calculated for each duplicated *PyyHsp70* gene using KaKs_Calculator [58].

### Promoter analysis of *PyyHSP70s*

The 5’ upstream regions, including 2000 bp DNA sequence upstream of each *PyyHSP70* gene, were subjected to the PlantCARE database (http://bioinformatics.psb.ugent.be/webtools/plantcare/html/) for a *cis*-elements scan.

### RNA-seq atlas analysis

To investigate the expression patterns of *PyyHSP70* genes in response to dehydration/rehydration treatments, the related RNA-sequencing (seq) data of *Py. yezoensis* were downloaded from NCBI under accession number PRJNA401507 [21]. The RNA-seq data of *Py.haitanensis* in dehydration/rehydration treatments were used to obtain expression patterns of *PyhHSP70* genes [20]. Expression heatmaps were constructed using R software and based on the FPKM values of gene expression in different treatments.

### RNA isolation and qRT-PCR analysis

By weighing the fresh weight and the dry weight of thalli, the absolute water content (AWC) of the thallus was calculated according to the methods described by Kim et al. (2009) [59]. Thalli produced under normal growth condition were harvested as the control group (AWC100). Before dehydration, the surface water of the thalli was removed by paper towels, and then the selected thalli were naturally dehydrated under 50 μmol photons m^−2^•s^−1^ at 8 ± 1 °C. The thalli samples were collected until the total water content decreased by 30% (AWC70), 50% (AWC50), and 80% (AWC20). After losing 80% water content, the samples were recovered in normal seawater for 30 min (AWC20_REH) [20, 21]. Three biological replicates were performed for each treatment. Samples were harvested and placed in liquid nitrogen before processing for gene expression analysis. Total RNA was extracted using the RNeasy Plant Mini Kit (OMEGA) according to the manufacturer’s instructions. Next, 1 μg total RNA was used to synthesize the first-strand cDNA using a HiScript® III RT SuperMix for qPCR (+ gDNA wiper) Kit (Vazyme Biotech). The qRT-PCR analysis was performed as described previously [60]. The expression levels of the ubiquitin-conjugating enzyme (UBC) and cystathionine gamma-synthase 1 (CGS1) genes were used as reference [61]. and the 2^−△△Ct^ method was used to calculate relative gene expression values. The sequences of the primers used are listed in Supplementary Table S5.

### Subcellular localization analysis of *PyyHsp70s*

To validate the prediction of subcellular localization, transient expression analyses were performed using a protoplast system based on the pBWA(V)HS-(PyyHSP70-1/ PyyHSP70-3)-GLosgfp vector. For two representative *PyyHSP70* genes, the full-length CDS without the stop codon was cloned into the pBWA(V)HS vector. Each CDS was fused in-frame to the N-terminus of the green fluorescent protein (GFP) coding sequence under the control of the CaMV 35S promoter. The primers used for PCR amplification of the full-length *HSP70* CDS are listed in Table S6. The vector with only *GFP* gene expressed was used as a control. The protoplasts used for transient expression analysis were extracted from *Arabidopsis* leaves and transformed by the polyethylene glycol (PEG) method [62]. Briefly, the *Arabidopsis* leaves was put into enzyme solution (1.5% (w/v) cellulose R10, 0.75% (w/v) macerozyme R10, 0.6 M mannitol, 10 mM MES, pH5.8) at 24 °C for 4 h with gentle shaking in the dark. After filtering through nylon mesh and washing two times with W5 solution (154 mM sodium chloride, 125 mM calcium chloride (CaCl_2_), 5 mM glucose, 2 mM KH_2_PO_4_, 2 mM MES, pH 5.7), protoplasts were resuspended in MMG solution (0.4 M mannitol, 15 mM magnesium chloride, 4 mM MES, pH 5.7) at a cell concentration of 2 × 10^5^ mL^−1^. Then, 10 μg of each plasmid sample was mixed with 100 μL protoplasts, followed by addition of 120 μL of freshly prepared PEG solution (40% (w/v) PEG4000, 0.6 M mannitol, and 100 mM CaCl_2_). The mixture was incubated at room temperature for 30 min in the dark, and then diluted gently with 1 mL W5 solution. After centrifugation at 300 rpm for 3 min, protoplasts were resuspended in 1 mL of W5 solution before incubating at 25 ℃ for 16 h and then observed using a Nikon Eclipse 80i fluorescence microscope. Respective excitation and emission wavelengths were 488 nm and 510 nm for the GFP signal, and 640 nm and 675 nm for the Chl signal.

## Supplementary Information

**Additional file 1: Table S1.** The corresponding amino acid number of the domains in HSP70 proteins.

**Additional file 2: Table S2.** Motifs of HSP70 Proteins.

**Additional file 3: Table S3.** The gene IDs of *HSP70*s.

**Additional file 4: Table S4.** Ka, Ks, and Ka/Ks values for duplicated ortholog pairs in *Py. yezoensis* and *Py. haitanensis*.

**Additional file 5: Table S5.** Gene primers designed for qRT-PCR.

**Additional file 6: Table S6.** Primers designed for subcellular localization analysis.

**Additional file 7: Figure S1.** Multiple sequence alignment all 15 PyyHSP70 proteins and the EcDNAK protein. Green box: ATPase domain; orange box: interdomain hinge; blue box: peptide-binding domain; red box: C-terminal sub-domain.

**Additional file 8: Figure S2.** Heatmap of the expression patterns of *PyhHSP70* genes under dehydration and rehydration treatments: absolute water content 100% (AWC100, control), absolute water content 70% (AWC70), absolute water content 20% (AWC20), rehydrated 30 min after 20% of water loss (AWC20_30min). The color bar represents log_2_ expression levels (FPKM). The tree (left) represents clustering result of *PyhHSP70*s’ expression patterns.

## Data Availability

RNA-seq data of *Py. yezoensis* in dehydration/rehydration treatments are available in NCBI under accession number PRJNA401507. RNA-seq data of *Py. haitanensis* in dehydration/rehydration treatments are available in NCBI under accession number PRJNA282903. All other datasets generated in this study are included as supplementary information of this article.

## Abbreviations

PyyHSP70: *Pyropia yezoensis* HSP70
CDS: Coding sequence
HMM: Hidden markov model
AWC: Absolute water content
REH: Rehydration
qRT-PCR: Quantitative real-time polymerase chain reaction
