# Supplementary material for: Genome-wide analysis of HSP70 gene superfamily in Pyropia yezoensis (Bangiales, Rhodophyta): identification, characterization and expression profiles in response to dehydration stress
Source: BMC Plant Biol. 2021 Sep 24;21:435. doi: 10.1186/s12870-021-03213-0 (PMC8464122; doi:10.1186/s12870-021-03213-0)
Supplement: Supplementary file 1 — Additional file 1: Table S1. The corresponding amino acid number of the domains in HSP70 proteins. [file 12870_2021_3213_MOESM1_ESM.docx]

Table S1. The corresponding amino acid number of the domains in HSP70 proteins.

| Protein ID | ATPase domain | Interdomain hinge | Peptide-binding domain | C-terminal sub-domain |
| --- | --- | --- | --- | --- |
| PyyHSP70-1 | 444 | 10 | 205 | 77 |
| PyyHSP70-2 | 432 | 10 | 124 | 0 |
| PyyHSP70-3 | 398 | 10 | 207 | 48 |
| PyyHSP70-4 | 439 | 10 | 207 | 17 |
| PyyHSP70-5 | 432 | 10 | 215 | 104 |
| PyyHSP70-6 | 79 | 10 | 217 | 80 |
| PyyHSP70-7 | 100 | 0 | 173 | 2 |
| PyyHSP70-8 | 226 | 2 | 194 | 91 |
| PyyHSP70-9 | 393 | 10 | 207 | 58 |
| PyyHSP70-10 | 432 | 10 | 215 | 104 |
| PyyHSP70-11 | 432 | 10 | 186 | 29 |
| PyyHSP70-12 | 390 | 10 | 414 | 119 |
| PyyHSP70-13 | 439 | 10 | 340 | 124 |
| PyyHSP70-14 | 340 | 10 | 215 | 104 |
| PyyHSP70-15 | 146 | 10 | 217 | 54 |
| EcDNAK | 385 | 10 | 199 | 44 |
