# Supplementary material for: Genome-wide analysis of HSP70 gene superfamily in Pyropia yezoensis (Bangiales, Rhodophyta): identification, characterization and expression profiles in response to dehydration stress
Source: BMC Plant Biol. 2021 Sep 24;21:435. doi: 10.1186/s12870-021-03213-0 (PMC8464122; doi:10.1186/s12870-021-03213-0)
Supplement: Supplementary file 2 — Additional file 2: Table S2. Motifs of HSP70 Proteins. [file 12870_2021_3213_MOESM2_ESM.docx]

Table S2. Motifs of HSP70 Proteins

| Motif | Width/aa | Sequences | Functions |
| --- | --- | --- | --- |
| Motif1 | 88 | KNVLVFDLGGGTFDVSJLTIEDGVFEVKATAGDTHLGGEDFDNRLVNHFAAEFKRKYKKDJSKBKRALRRLRTACEKAKRTLSSTTQT | In the ATPase domain; |
| Motif2 | 47 | KBAVVTVPAYFNDSQRQATKDAGTIAGLNVLRIINEPTAAAJAYGLD | In the ATPase domain; |
| Motif3 | 49 | VYEGERARTKDNNLLGKFDLSGIPPAPRGVPQIEVTFDIDANGILNVSA | In the substrate peptide binding domain |
| Motif4 | 50 | LLLDVTPLSLGIETAGGVMTKLIPRNTTIPTKKSQVFSTYADNQPGVLIQ | In the substrate peptide binding domain |
| Motif5 | 36 | IGDAAKNQAAMNPENTVFDVKRLIGRRFSDPSVQRD | In the ATPase domain; |
| Motif6 | 23 | LNKSINPDEAVAYGAAVQAAILS | In the ATPase domain; |
| Motif7 | 44 | EPVEKVLKDAKLDKSQVDEIVLVGGSTRIPKVQQLLKDFFNGKE | In the ATPase domain; |
| Motif8 | 47 | DKGRLSKEEIEKMVQEAEKYKAEDEEVKKKVEAKNGLENYAYNLKNT | In the substrate peptide binding domain |
| Motif9 | 22 | NGGVEIIANDZGNRTTPSYVAF | In the ATPase domain; |
| Motif10 | 31 | IESLFDGVDFYSTJTRAKFEELCADLFRRTL | In the ATPase domain; |
| Motif11 | 31 | YKGETKQFSPEZISAMVLSKMKETAEAYLGK | In the ATPase domain; |
| Motif12 | 16 | IGIDLGTTYSCVAVWR | In the ATPase domain; |

Motif numbers corresponded to the motifs in Fig. 2.
