# Supplementary material for: Genome-wide analysis of HSP70 gene superfamily in Pyropia yezoensis (Bangiales, Rhodophyta): identification, characterization and expression profiles in response to dehydration stress
Source: BMC Plant Biol. 2021 Sep 24;21:435. doi: 10.1186/s12870-021-03213-0 (PMC8464122; doi:10.1186/s12870-021-03213-0)
Supplement: Supplementary file 3 — Additional file 3: Table S3. The gene IDs of HSP70s. [file 12870_2021_3213_MOESM3_ESM.docx]

Table S3. The gene IDs of *HSP70*s.

| Pyropia haitanensis | | | Porphyra umbilicalis | | | Chondrus crispus | | | Galdieria sulphuraria | | | Porphyridium purpureum | | | Others | | | |
| --- | --- | --- | --- | --- | --- | --- | --- | --- | --- | --- | --- | --- | --- | --- | --- | --- | --- | --- |
| No. | Gene ID | Chr.:location coordinates  (5’-3’ ) | No. | Gene ID | Chr.:location coordinates  (5’-3’ ) | No. | Gene ID | Chr.:location coordinates  (5’-3’ ) | No. | Gene ID | Chr.:location coordinates  (5’-3’ ) | No. | Gene ID | Chr.:location coordinates  (5’-3’ ) | Gene ID | Accession | Gene ID | Accession |
| PyhHSP70-1 | ph09082 | SDUX01000001.1:1002260-1009127(+) | PorphyrauHSP70-1 | OSX71346.1 | KV919144.1:38709-40748(+) | CcHSP70-1 | XP_005711716.1 | NW_005178804.1:213731-215749(+) | GsHSP70-1 | XP_005702886.1 | NW_005178408.1:16822-20940(-) | PorphyripHSP70-1 | KAA8491802.1 | VRMN01000011.1:124187-126223(+) | AtHSP70-1 | CAB85987.1 | AtHSP70-14 | AAG52240.1 |
| PyhHSP70-2 | ph09665 | SDUX01000002.1:5668537-5665820(-) | PorphyrauHSP70-2 | OSX73076.1 | KV919021.1:102315-104988(+) | CcHSP70-2 | XP_005712413.1 | NW_005178881.1:117007-119123(-) | GsHSP70-2 | XP_005703010.1 | NW_005178411.1:43811-47249(+) | PorphyripHSP70-2 | KAA8492600.1 | VRMN01000009.1:514359-517061(+) | AtHSP70-2 | CAB85986.1 | AtHSP70-15 | AAG52244.1 |
| PyhHSP70-3 | ph02484 | SDUX01000003.1:1161678-1159035(-) | PorphyrauHSP70-3 | OSX74071.1 | KV918965.1:43059-45092(+) | CHSP70-3 | XP_005713917.1 | NW_005179024.1:105166-107459(-) | GsHSP70-3 | XP_005703818.1 | NW_005178425.1:86151-88591(+) | PorphyripHSP70-3 | KAA8492858.1 | VRMN01000008.1:311980-314715(-) | AtHSP70-3 | AAF14038.1 | AtHSP70-16 | AAD30257.1 |
| PyhHSP70-4 | ph10339 | SDUX01000004.1:2296087-2298965(+) | PorphyrauHSP70-4 | OSX74148.1 | KV918961.1:118453-121044(-) | CcHSP70-4 | XP_005714152.1 | NW_005179036.1:201472-203674(-) | GsHSP70-4 | XP_005704870.1 | NW_005178438.1:104918-107280(-) | PorphyripHSP70-4 | KAA8492910.1 | VRMN01000008.1:440764-442731(-) | AtHSP70-4 | BAB02269.1 | AtHSP70-18 | AAG51503.1 |
| PyhHSP70-5 | ph00654 | SDUX01000004.1:5710823-5705713(-) | PorphyrauHSP70-5 | OSX74808.1 | KV918930.1:111768-113047(-) | CcHSP70-5 | XP_005716615.1 | NW_005179154.1:29302-32255(+) | GsHSP70-5 | XP_005704871.1 | NW_005178438.1:104786-107280(-) | PorphyripHSP70-5 | KAA8497180.1 | VRMN01000002.1:1784954-1786969(+) | AtHSP70-5 | AAF18501.1 | ScSSA1 | P10591 |
| PyhHSP70-6 | ph10775 | SDUX01000007.1:1788009-1792503(+) | PorphyrauHSP70-6 | OSX76008.1 | KV918883.1:165937-167820(+) | CcHSP70-6 | XP_005717406.1 | NW_005179199.1:107461-110466(-) | GsHSP70-6 | XP_005705015.1 | NW_005178440.1:85041-87005(+) |  |  |  | AtHSP70-6 | CAB45063.1 | ScSSA2 | P10592 |
| PyhHSP70-7 | ph09518 | SDUX01000007.1:5975265-5977304(+) |  |  |  | CcHSP70-7 | XP_005718499.1 | NW_005179300.1:25760-27944(+) | GsHSP70-7 | XP_005707456.1 | NW_005178464.1:43121-45189(+) |  |  |  | AtHSP70-7 | BAA97012.1 | ScSSA3 | S36753 |
| PyhHSP70-8 | ph08047 | SDUX01000120.1:13336-14912(+) |  |  |  | CcHSP70-8 | YP_007627389.1 | NC_020795.1:82554-84428(+) | GsHSP70-8 | XP_005707633.1 | NW_005178465.1:102051-104191(+) |  |  |  | AtHSP70-8 | AAD15393.1 | ScSSA4 | B36590 |
|  |  |  |  |  |  |  |  |  |  |  |  |  |  |  | AtHSP70-9 | CAB37531.1 | ScSSC1 | M27229 |
|  |  |  |  |  |  |  |  |  |  |  |  |  |  |  | AtHSP70-10 | CAB89371.1 | ScSSE1 | P32589 |
|  |  |  |  |  |  |  |  |  |  |  |  |  |  |  | AtHSP70-11 | AAF88019.1 | ScSSE2 | P32590 |
|  |  |  |  |  |  |  |  |  |  |  |  |  |  |  | AtHSP70-12 | BAB08435.1 | ScSSH1 | S44545 |
|  |  |  |  |  |  |  |  |  |  |  |  |  |  |  | AtHSP70-13 | AAB70400.1 | EcDNAK | WP_000516135.1 |
