# Supplementary material for: Genome-wide analysis of HSP70 gene superfamily in Pyropia yezoensis (Bangiales, Rhodophyta): identification, characterization and expression profiles in response to dehydration stress
Source: BMC Plant Biol. 2021 Sep 24;21:435. doi: 10.1186/s12870-021-03213-0 (PMC8464122; doi:10.1186/s12870-021-03213-0)
Supplement: Supplementary file 4 — Additional file 4: Table S4. Ka, Ks, and Ka/Ks values for duplicated ortholog pairs in Py. yezoensis and Py. haitanensis. [file 12870_2021_3213_MOESM4_ESM.docx]

Table S4. Ka, Ks, and Ka/Ks values for duplicated ortholog pairs in *Py. yezoensis* and *Py. haitanensis*

| Seq 1 | Seq 2 | Ka | Ks | Ka/Ks |
| --- | --- | --- | --- | --- |
| *PyyHSP70-1* | *PyhHSP70-2* | 0.0226 | 0.6051 | 0.0373 |
| *PyyHSP70-3* | *PyhHSP70-3* | 0.9204 | 1.4176 | 0.6493 |
| *PyyHSP70-4* | *PyhHSP70-5* | 0.0088 | 1.0314 | 0.0085 |
| *PyyHSP70-9* | *PyhHSP70-4* | 0.0198 | 0.9784 | 0.0203 |
| *PyyHSP70-11* | *PyhHSP70-7* | 0.0087 | 0.5679 | 0.0153 |
| *PyyHSP70-12* | *PyhHSP70-6* | 0.0465 | 0.6615 | 0.0703 |
| *PyyHSP70-13* | *PyhHSP70-1* | 0.0389 | 0.4875 | 0.0798 |
