# Supplementary material for: Genome-wide analysis of HSP70 gene superfamily in Pyropia yezoensis (Bangiales, Rhodophyta): identification, characterization and expression profiles in response to dehydration stress
Source: BMC Plant Biol. 2021 Sep 24;21:435. doi: 10.1186/s12870-021-03213-0 (PMC8464122; doi:10.1186/s12870-021-03213-0)
Supplement: Supplementary file 5 — Additional file 5: Table S5. Gene primers designed for qRT-PCR. [file 12870_2021_3213_MOESM5_ESM.docx]

Table S5. Gene primers designed for qRT-PCR.

| **Primers** | **Primer sequences (5' to 3')** |
| --- | --- |
| CGS1-F | CTACGGACACCAAGAAACG |
| CGS1-R | CTCGGTTGGCTGGGTAA |
| UBC-F | TCACAACGAGGATTTACCACC |
| UBC-R | GAGGAGCACCTTGGAAACG |
| PyyHSP70-1-F | TACCAACAAGCAGCAGAGCAT |
| PyyHSP70-1-R | TCGAGTAGAGAAGCGAGTCCG |
| PyyHSP70-3-F | CCAAGGACTGTGGTCTGCTC |
| PyyHSP70-3-R | GTAATCTTGTTCTGCTTGCCG |
| PyyHSP70-4-F | GGTGAAGGAGTTCTTTGGCG |
| PyyHSP70-4-R | GGCGTCTTCATGCTCGTGTC |
| PyyHSP70-8-F | CTCATCGGTCGCCACTTCTC |
| PyyHSP70-8-R | CTGGCACTCAATCTGCACAAA |
| PyyHSP70-9-F | CATCACGAACGACAAGGGGC |
| PyyHSP70-9-R | GACGTTGTACAGATAGCCCTCCA |
| PyyHSP70-11-F | CAACCGTATCACCCCATCGT |
| PyyHSP70-11-R | GACTGACACCTCCACCATCG |
| PyyHSP70-12-F | TATCTGTGTTCTCCGCTCGC |
| PyyHSP70-12-R | GGACTTGTCGTCATCAGCCA |
| PyyHSP70-13-F | GCCAAGACCATCATCCATCG |
| PyyHSP70-13-R | CGTTCGCAGCAGCATTCAG |
