# Supplementary material for: Genome-wide analysis of HSP70 gene superfamily in Pyropia yezoensis (Bangiales, Rhodophyta): identification, characterization and expression profiles in response to dehydration stress
Source: BMC Plant Biol. 2021 Sep 24;21:435. doi: 10.1186/s12870-021-03213-0 (PMC8464122; doi:10.1186/s12870-021-03213-0)
Supplement: Supplementary file 6 — Additional file 6: Table S6. Primers designed for subcellular localization analysis. [file 12870_2021_3213_MOESM6_ESM.docx]

Table S6. Primers designed for subcellular localization analysis

| Primers | Primer sequences |
| --- | --- |
| PyyHSP70-1 (+) | cagtTTTCGCAGCATCTAACGAGCTCTTCGctacatggcgtctgcagtgagcgc |
| PyyHSP70-1 (-) | cagtTTTCGCAGCATCTAACGAGCTCTTCGtacactaggcagagctcccgccgc |
| PyyHSP70-3 (+) | cagtCACCTGCaaaacaacatggccgacaagaaggacgt |
| PyyHSP70-3 (-) | cagtCACCTGCaaaatacagtcgatatcctccacggtaa |
