# Supplementary material for: Genome-wide analysis of HSP70 gene superfamily in Pyropia yezoensis (Bangiales, Rhodophyta): identification, characterization and expression profiles in response to dehydration stress
Source: BMC Plant Biol. 2021 Sep 24;21:435. doi: 10.1186/s12870-021-03213-0 (PMC8464122; doi:10.1186/s12870-021-03213-0)
Supplement: Supplementary file 7 — Additional file 7: Figure S1. Multiple sequence alignment all 15 PyyHSP70 proteins and the EcDNAK protein. Green box: ATPase domain; orange box: interdomain hinge; blue box: peptide-binding domain; red box: C-terminal sub-domain. [file 12870_2021_3213_MOESM7_ESM.docx]

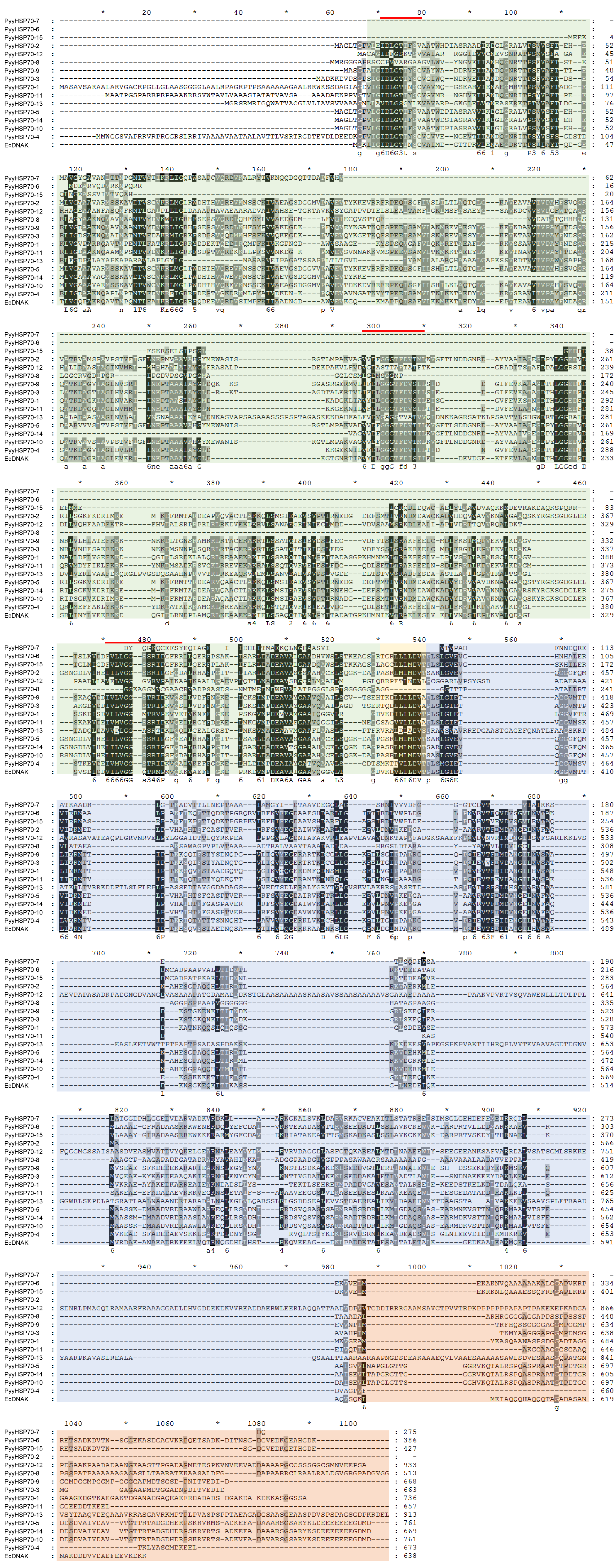


Fig.S1 Multiple sequence alignment all 15 PyyHSP70 proteins and the EcDNAK protein. Green box: ATPase domain; orange box: interdomain hinge; blue box: peptide-binding domain; red box: C-terminal sub-domain.
