# Supplementary material for: Genome-wide analysis of HSP70 gene superfamily in Pyropia yezoensis (Bangiales, Rhodophyta): identification, characterization and expression profiles in response to dehydration stress
Source: BMC Plant Biol. 2021 Sep 24;21:435. doi: 10.1186/s12870-021-03213-0 (PMC8464122; doi:10.1186/s12870-021-03213-0)
Supplement: Supplementary file 8 — Additional file 8: Figure S2. Heatmap of the expression patterns of PyhHSP70 genes under dehydration and rehydration treatments: absolute water content 100% (AWC100, control), absolute water content 70% (AWC70), absolute water content 20% (AWC20), rehydrated 30 min after 20% of water loss (AWC20_30min). The color bar represents log2 expression levels (FPKM). The tree (left) represents clustering result of PyhHSP70s’ expression patterns. [file 12870_2021_3213_MOESM8_ESM.docx]

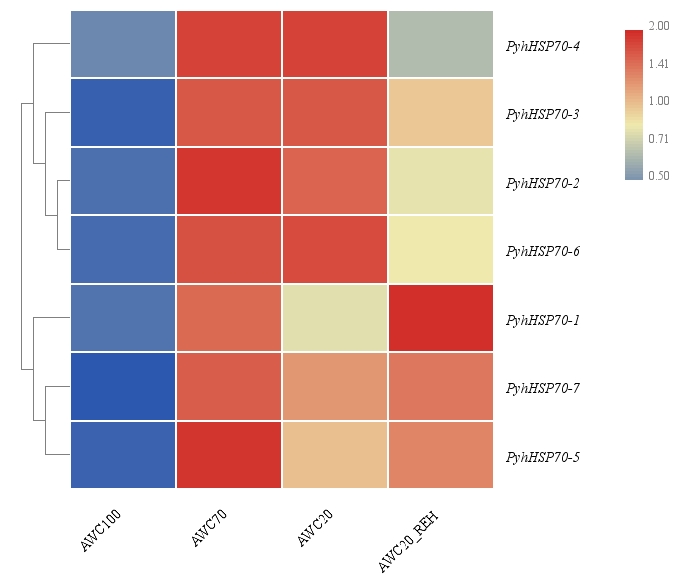


Fig.S2 Heatmap of the expression patterns of *PyhHSP70* genes under dehydration and rehydration treatments: absolute water content 100% (AWC100, control), absolute water content 70% (AWC70), absolute water content 20% (AWC20), rehydrated 30 min after 20% of water loss (AWC20_30min). The color bar represents log_2_ expression levels (FPKM). The tree (left) represents clustering result of *PyhHSP70*s’ expression patterns.
